# Recircumscription of *Begonia* sect. *Baryandra* (Begoniaceae): evidence from molecular data

**DOI:** 10.1186/1999-3110-54-38

**Published:** 2013-09-24

**Authors:** Rosario Rivera Rubite, Mark Hughes, Grecebio JD Alejandro, Ching-I Peng

**Affiliations:** 1 Department of Biology, College of Arts and Sciences, University of the Philippines Manila, Padre Faura, Manila, 1000 Philippines; 2 Royal Botanic Garden Edinburgh, 20a Inverleith Row, Edinburgh, EH3 5LR UK; 3 College of Science and Research Centre for the Natural and Applied Sciences, University of Santo Tomas, España, Manila, 1015 Philippines; 4 Biodiversity Research Center, Academia Sinica, Taipei, 115 Taiwan

**Keywords:** *Begonia*, Classification, Section, Phylogeny, Taxonomy

## Abstract

**Background:**

*Begonia* sect. *Diploclinium* is a ‘dust-bin’ section for species retaining pleisiomorphic characters and lacking novel synapomorphic characters used to delimit other Asian sections in *Begonia*. Part of this large and polymorphous *section* is transferred to *Begonia* sect. *Baryandra* in a move towards a more natural classification for the genus.

**Results:**

Phylogenetic analysis of nuclear ribosomal ITS DNA sequences show a strongly supported monophyletic group containing Philippine and Bornean species previously in *Begonia* sect. *Diploclinium*, and the type of *Begonia* sect. *Baryandra*, *B. oxysperma*. This clade forms the basis for the now much-expanded *Begonia* sect. *Baryandra*, which as defined here contains 49 species and has its centre of diversity in the Philippines.

**Conclusions:**

A natural classification for a much expanded *Begonia* sect. *Baryandra* has been provided. This paper highlights the feasibility of moving towards a natural classification of Asian *Begonia* step by step as information comes to light through building upon previous framework phylogenies with denser sampling.

**Electronic supplementary material:**

The online version of this article (doi:10.1186/1999-3110-54-38) contains supplementary material, which is available to authorized users.

## Background

Large genera are unwieldy units that make aspects of their biogeography, ecology or morphology difficult to discuss without dividing them into smaller groupings. In large genera, infrageneric ranks take over the function of the genus as it is used for less species-rich groups; sub-genera or sections are able to divide large genera into more manageable units for discussing and communicating biological information. Ideally, sub-generic taxa will have a biological reality, e.g. be monophyletic, to make them meaningful, as opposed to being groupings of phenetically similar but possibly not closely related species. Sub-generic taxa have another use in that they allow one to freely move species from one higher taxon to another without having to formally publish new combinations and leaving an untidy nomenclatural trail. This is particularly relevant for using the rank of section in *Begonia*. Following the reduction of the many genera formerly recognised by Klotzsch (1855), the delimitation of the genus *Begonia* is currently uncontroversial, however there is still a lot of uncertainty about which sections some species belong to (Doorenbos et al., 1998). Although much work remains to be done in terms of understanding the relationships of the ca. 1600 species of *Begonia*, enough phylogenetic evidence is available (Forrest et al., 2005; Tebbitt, 2006; Thomas et al., 2011; this study) to enable us to start making some changes in sectional delimitation. This means we can begin to move away from a classification based on making-do with sections which in some cases are not only polyphyletic, but also scarcely phenetically similar assemblages such as *Begonia* sect. *Diploclinium* (Doorenbos et al., 1998; Thomas et al., 2011).

**Figure 1:**
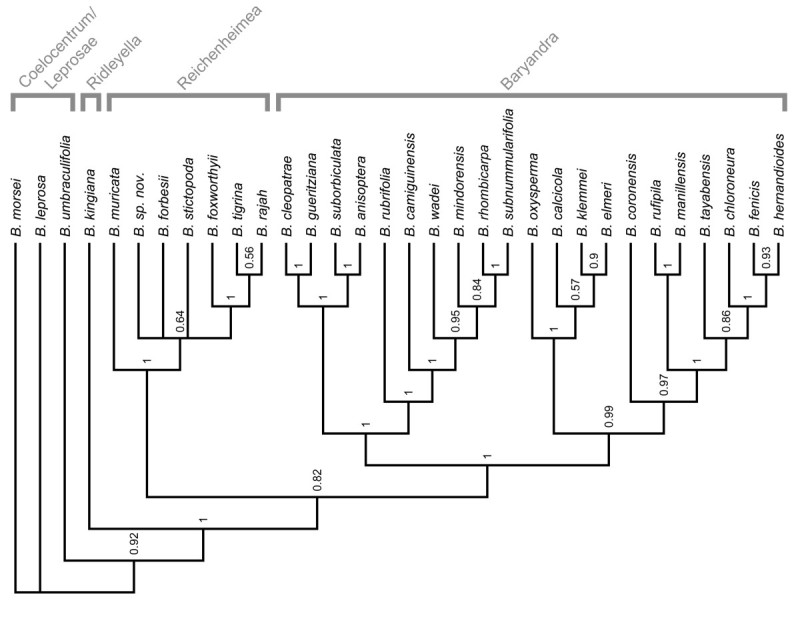
**Cladogram of a Bayesian phylogenetic analysis of nuclear ribosomal internal transcribed spacer DNA sequences from** ***Begonia*** **sect.** ***Coelocentrum*** **,** ***Begonia*** **sect.** ***Leprosae*** **(outgroups),** ***Begonia*** **sect.** ***Ridleyella*** **,** ***Begonia*** **sect.** ***Reichenheimea*** **and 21 out of 55 species in** ***Begonia*** **sect.** ***Baryandra*** **.** Numbers above the nodes show posterior probability.

*Begonia* sect. *Diploclinium* is one of the most problematic sections in the genus, Doorenbos et al., (1998) referred to it as “a show-case of the difficulties one meets when trying to delimit sections”. The lack of distinguishing characters for the section has been highlighted by Shui et al., (2002) who note its similarity to not only other Asian sections but also the New World *Begonia* sect. *Begonia* and *Begonia* sect. *Knesebeckia*. Based on an analysis of how morphological characters used in defining *Begonia* sections evolve across a chloroplast DNA phylogeny (Thomas et al., 2011), it has become clear that *Begonia* sect. *Diploclinium* is a ‘dust-bin’ section for species retaining pleisiomorphic characters and lacking novel synapomorphic characters used to delimit other Asian sections. The same study shows that its type species, *Begonia grandis*, diverges at the base of a grade of tuberous species from continental Asia; nested within this grade are *Begonia* sect. *Sphenanthera* and *Begonia* sect. *Platycentrum*. Philippine species assigned to *Begonia* sect. *Diploclinium* do not fall within this clade, but are nested within a predominantly Malesian clade and sister to *Begonia* (Tebbitt, sect. *Reichenheimea*^2006^; Thomas et al., 2011). The placement of the Philippine species in *Begonia* sect. *Diploclinium* is based solely on the presence of bifid placentae, a character found in the majority of the species of *Begonia* (Forrest et al., 2005). Following a review of morphology (Rubite, 2012) and additional phylogenetic investigations with increased taxon sampling (Figure 1), we here formally move a group of largely Philippine species previously in *Begonia* sect. *Diploclinium* into a much expanded *Begonia* sect. *Baryandra*, previously containing only *Begonia oxysperma*.

## Methods

DNA sequences of the nuclear ribosomal internal transcribed spacers ITS1 and ITS2 plus the 5.8S gene were obtained from samples of *Begonia* sect. *Coelocentrum*, *Begonia* sect. *Leprosae* (outgroups), *Begonia* sect. *Ridleyella*, *Begonia* sect. *Reichenheimea* and 21 out of 49 species in *Begonia* sect. *Baryandra* as recircumscribed here using the methods of Forrest et al., (2005) (Table 1). Considering both morphological (Doorenbos et al., 1998) and molecular (Tebbitt et al., 2006; Thomas et al., 2011) data the closest relatives of the Philippine species in *Begonia* sect. *Baryandra* are species in *Begonia* sect. *Reichenheimea* from the Sunda Shelf. The sampling was designed to test the monophyly of these two sections. In addition, morphologically divergent species of *Begonia* sect. *Baryandra* were sampled (*B. coronensis*, which has entire placentae; *B. anisoptera*, *B. gueritziana* and *B. suborbiculata*, which have 2-locular fruit), as was the type of *Begonia* sect. *Baryandra*, *B. oxysperma*. The type of *Begonia* sect. *Diploclinium*, *B. grandis*, was not included as the sequences are too divergent to align unambiguously with the other samples, and previous work (Thomas et al., 2011) has shown that it does not belong to the ingroup. Sequences were aligned manually, and a phylogenetic analysis was carried out using MrBayes (Ronquist et al., 2012) using a GTR + G model of sequence evolution, 10 million generations and a burn-in of 25%.

**Table 1:** **Species included in molecular phylogenetic analysis with GenBank accession numbers and voucher information**

Species	GenBank accession	Voucher
*B. anisoptera*	JX656720	Rubite R479 (PNH)
*B. calcicola*	JX656708	Peng P20761 (HAST)
*B. camiguinensis*	JX656721	Rubite R506 (PNH)
*B. chloroneura*	AF485134	Forrest 128 (E)
*B. cleopatrae*	AF485133	Forrest 127 (E)
*B. coronensis*	JX656715	Rubite R323 (PNH)
*B. elmeri*	JX656714	Rubite R319 (PNH)
*B. fenicis*	JX678218	Peng P18366 (HAST)
*B. forbesii*	JX656704	Peng P22685 (HAST)
*B. foxworthyii*	JX656702	Peng P22721 (HAST)
*B. gueritziana*	JX678217	Peng 21976 (HAST)
*B. hernandioides*	JX656707	Rubite R106 (PNH)
*B. kingiana*	AF485139	Forrest 133 (E)
*B. klemmei*	JX656709	Rubite R182 (PNH)
*B. leprosa*	AY753722	Tebbitt 94 (BKL)
*B. manillensis*	JX656713	Rubite R304 (PNH)
*B. mindorensis*	JX656717	Rubite R354 (PNH)
*B. morsei*	AF485130	No voucher
*B. muricata*	AY753725	Hoover 901 (A)
*B. oxysperma*	JX656710	Rubite R213 (PNH)
*B. rajah*	AF485136	Forrest 130 (E)
*B. rhombicarpa*	JX656719	Rubite R419 (PNH)
*B. rubrifolia*	JX656711	Rubite R234 (PNH)
*B. rufipila*	JX656712	Rubite R265 (PNH)
*B. sp. nov.*	JX656701	Girmansyah & Hughes DEDEN1490 (E)
*B. stictopoda*	JX656705	Hughes MH1409 (E)
*B. subnummularifolia*	JX656722	No voucher
*B. suborbiculata*	JX656716	Rubite R353 (PNH)
*B. tayabensis*	JX656718	Rubite R360 (PNH)
*B. tigrina*	JX656703	Peng P22720 (HAST)
*B. umbraculifolia*	JF976054	Shui et al., SYM-B2005-086-sample2 (KUN)
*B. wadei*	JX656706	Rubite 699 (PNH)

## Results

The phylogeny obtained (Figure 1) shows a strongly supported monophyletic group containing Philippine and Bornean species previously in *Begonia* sect. *Diploclinium*, and the type of *Begonia* sect. *Baryandra*, *B. oxysperma*. This clade forms the basis for the now much-expanded *Begonia* sect. *Baryandra*, which as defined here contains 49 species and has its centre of diversity in the Philippines.

## Discussion

*Begonia* sect. *Baryandra* is morphologically most similar to (and phylogenetically closest to) *Begonia* sect. *Reichenheimea* as represented by species from Peninsular Malaysia and Sumatra, differing in having 2 placentae per locule (not 1) and boat-shaped, entire, sheathing bracts (not flat-ovate, minutely fimbriate, reflexed bracts). We reviewed the morphology of all Malesian species in *Begonia* (Hughes, sect. *Diploclinium*^2008^) to determine if they should be placed in *Begonia* sect. *Baryandra*, and several were found not to have affinity with sect. *Baryandra* as delimited here. *Begonia longovillosa* from the Philippines is known only from a very short protologue and is here considered unplaced to section. From Peninsular Malaysia there are two species of *Begonia* sect. *Diploclinium* (Kiew, that do not match sect. *Baryandra*^2005^). *Begonia jayaensis* has bracts fringed with glandular hairs, with 1 or 2 5-tepalled carpellate flowers borne at the base of a larger staminate inflorescence and is probably referable to *Begonia* sect. *Petermannia*. *Begonia lowiana* is a caulescent species of uncertain affinity with the rest of *Begonia* sect. *Diploclinium*, and not allied to any Philippine taxa. From Sumatra there are three *Begonia* sect. *Diploclinium* species recorded; *Begonia sublobata* Jack has entire placentae and has been transferred to *Begonia* sect. *Reichenheimea* (Hughes and Girmansyah, 2011). The other two species, *Begonia hasskarliana* and *B. ionophylla*, are poorly known and information on their placentation is lacking; given their geographic location they likely also belong to *Begonia* sect. *Reichenheimea* and do not have affinity with *Begonia* sect. *Baryandra*.

There are four remaining Bornean species of *Begonia* sect. *Diploclinium* that we have not transferred to *Begonia* sect. *Baryandra*. *Begonia piring* has paired carpellate flowers with 5 tepals borne beneath a larger staminate inflorescence and hence is likely affiliated with *Begonia* sect. *Petermannia*. *Begonia havilandii* is known only from the protologue, but the description includes a paniculate inflorescence, carpellate flowers with 6 tepals and toothed bracts with glandular hairs, which are again suggestive of sect. *Petermannia* with an affinity to other creeping species such as *B. humericola* Sands. *Begonia sabahensis* and *B. calcarea* are closely allied species with orange and yellow flowers respectively, borne on separate umbellate staminate and unifloral carpellate inflorescences. The carpellate flowers have 5 tepals, and the staminate flowers have lax sessile stamens; it is not immediately clear which section these belong to, but it is obvious they have no affinity to *Begonia* sect. *Baryandra*.

With the exception of *Begonia sharpeana*, all of the other six species of *Begonia* sect. *Diploclinium* from New Guinea differ considerably from *Begonia* sect. *Baryandra* in either being tuberous, having a highly reduced number of stamens, lax and sessile androecia, or spurred fruit. These and the other species above which we have not transferred to *Begonia* sect. *Baryandra* we leave in *Begonia* sect. *Diploclinium* until further molecular or morphological data are known. A full recircumscription of *Begonia* sect. *Diploclinium* is beyond the scope of this paper, and will involve enlarging more of the currently described sections of the genus and will likely require several more to be proposed. Much more sampling across Asia is needed before this can be done with confidence, and this is likely to take some time.

### Taxonomic treatment

*Begonia* sect. *Baryandra* A.DC. **Type species:** *Begonia oxysperma* A.DC.

Herbs, rhizomatous (rarely lianescent: *B. oxysperma*), lacking an erect stem. Tubers absent. Stipules persistent, entire, ovate to lanceolate apex with a filiform extension, glabrous or hairy, adaxial surface often keeled. Leaves alternate, petiolate; petioles terete, glabrous to densely hairy; lamina usually asymmetric, basifixed or peltate, venation palmate-pinnate, abaxially with hairs on the veins, adaxially usually glabrous. Inflorescences axillary, dichasial, bisexual cymes, with staminate flowers basal and carpellate flowers distal, protandrous; bracts boat-shaped, entire, usually sheathing developing buds, caducous. Staminate flowers with 4 free perianth segments; androecium actinomorphic, filaments fused below into a short column, anthers oblong, shorter than the filaments, dehiscing with laterally positioned short slits. Carpellate flowers with 4 (rarely 5: *B. chloroneura*, *B. fenicis*, *B. hernandioides*, *B. tayabensis*) perianth segments; ovary with 3 equal or unequal wings, locules (2 or) 3, placentation axillary, placental branches 2 per locule (rarely 1: *B. coronensis*), ovules present between placental branches; styles 3, persistent or caducous in fruit, stigma in a spiralled band. Fruit pendulous or recurved at maturity.

BORNEO. ***Begonia diwolii*** Kiew, ***Begonia gueritziana*** Gibbs, ***Begonia subnummularifolia*** Merr.

PHILIPPINES. ***Begonia acclivis*** Coyle, ***Begonia acuminatissima*** Merr. (synonym: *Begonia camiguinensis* Elmer), ***Begonia alba*** Merr., ***Begonia alvarezii*** Merr., ***Begonia angilogensis*** Merr., ***Begonia anisoptera*** Merr., ***Begonia biliranensis*** Merr., ***Begonia blancii*** M.Hughes, ***Begonia calcicola*** Merr., ***Begonia castilloi*** Merr., ***Begonia chloroneura*** P.Wilkie & Sands, ***Begonia cleopatrae*** Coyle, ***Begonia collisiae*** Merr., ***Begonia colorata*** Warb., ***Begonia copelandii*** Merr., ***Begonia coronensis*** Merr., ***Begonia elmeri*** Merr., ***Begonia fenicis*** Merr., ***Begonia gitingensis*** Elmer, ***Begonia gutierrezii*** Coyle, ***Begonia hernandioides*** Merr., ***Begonia isabelensis*** Quisumb. & Merr., ***Begonia klemmei*** Merr., ***Begonia lancilimba*** Merr., ***Begonia longinoda*** Merr., ***Begonia longiscapa*** Warb., ***Begonia luzonensis*** Warb., ***Begonia manillensis*** A.DC., ***Begonia mindorensis*** Merr. (synonyms: *Begonia pinamalayensis* Merr., *Begonia sordidissima* Elmer), ***Begonia neopurpurea*** L.B.Sm. & Wassh., ***Begonia obtusifolia*** Merr., ***Begonia oxysperma*** A.DC., ***Begonia parva*** Merr., ***Begonia rhombicarpa*** A.DC. (synonyms: *Begonia merrillii* Warb., *Begonia nigritarum* Steud. ex Merr., *Begonia rhombicarpa* var. *lobbii* A.DC.), ***Begonia rubitae*** M.Hughes, ***Begonia rubrifolia*** Merr., ***Begonia rufipila*** Merr., ***Begonia serpens*** Merr., ***Begonia suborbiculata*** Merr., ***Begonia tayabensis*** Merr., ***Begonia trichocheila*** Warb., ***Begonia vanoverberghii*** Merr., ***Begonia wadei*** Merr. & Quisumb., ***Begonia wilkiei*** Coyle, ***Begonia woodii*** Merr.

NEW GUINEA. ***Begonia sharpeana*** F.Muell.

## Conclusion

A natural classification for a much expanded *Begonia* sect. *Baryandra* has been provided. A total of 49 species is now considered to belong to the section, which has its centre of diversity in the Philippines but also with some representatives in Borneo and New Guinea. This paper highlights the feasibility of moving towards a natural classification of Asian *Begonia* step by step as information comes to light through building upon previous framework phylogenies with denser sampling.

## Authors’ original submitted files for images

Below are the links to the authors’ original submitted files for images. Authors’ original file for figure 1
