# Supplementary figures and images for: Recircumscription of Begonia sect. Baryandra (Begoniaceae): evidence from molecular data
Source: Bot Stud. 2013 Sep 24;54:38. doi: 10.1186/1999-3110-54-38 (PMC5432953; doi:10.1186/1999-3110-54-38)

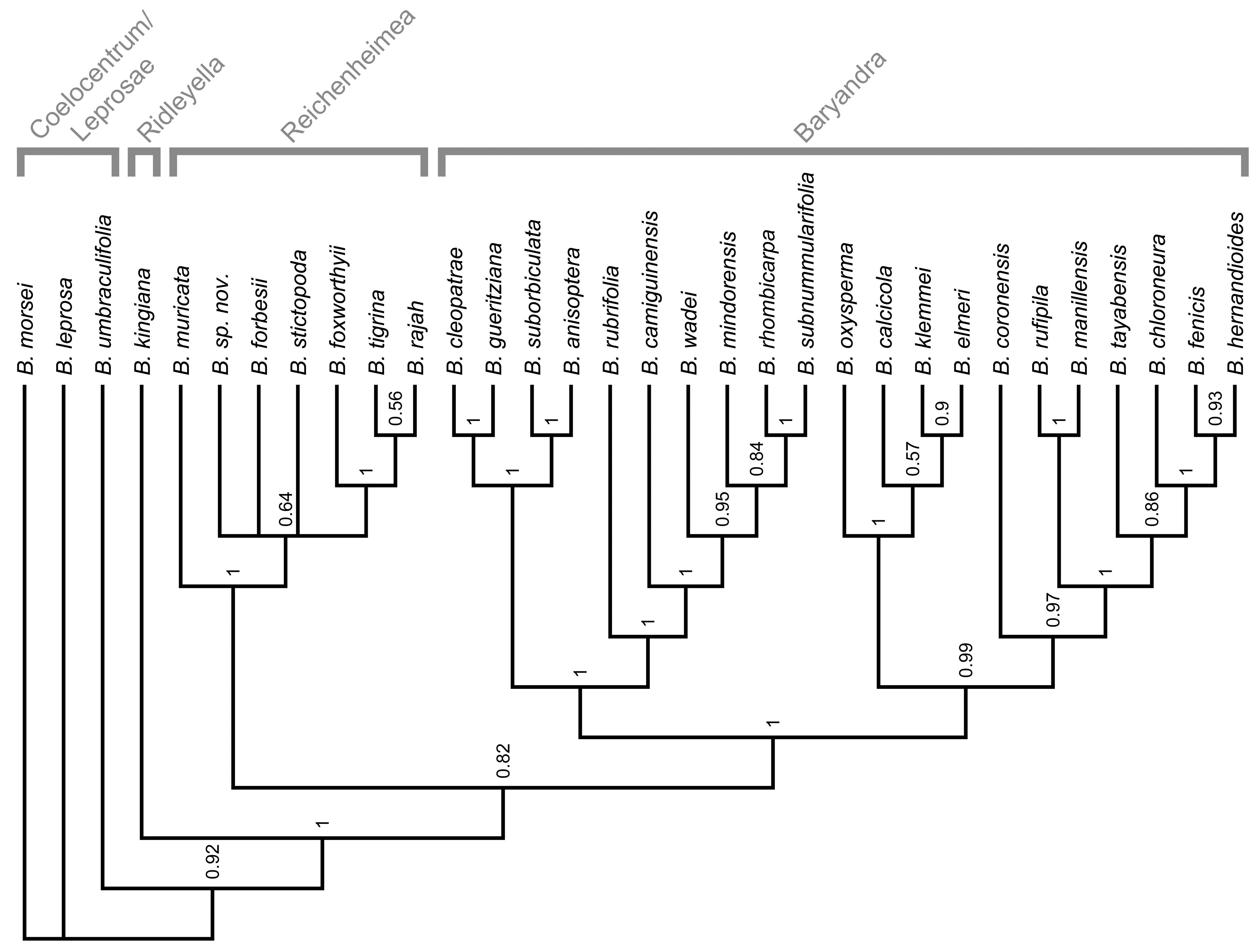

Supplement: Supplementary file 1 — Authors’ original file for figure 1 [file 40529_2013_34_MOESM1_ESM.tiff]
